# Marine conservation: towards a multi-layered network approach

**DOI:** 10.1098/rstb.2019.0459

**Published:** 2020-11-02

**Authors:** Ute Jacob, Andrew Beckerman, Mira Antonijevic, Laura E. Dee, Anna Eklöf, Hugh P. Possingham, Ross Thompson, Thomas J. Webb, Benjamin S. Halpern

**Affiliations:** 1 Helmholtz-Institute for Functional Marine Biodiversity at the University of Oldenburg (HIFMB), Ammerländer Heerstrasse 231, 26129 Oldenburg, Germany; 2 Alfred Wegener Institute, Helmholtz Centre for Polar and Marine Research, Am Handelshafen 12, 27570 Bremerhaven, Germany; 3 Department of Animal and Plant Sciences, University of Sheffield, Sheffield, UK; 4 ach and krach GmbH, Branddesign, Grindelberg 17, Hamburg, Germany; 5 Department of Ecology and Evolutionary Biology, University of Colorado, Boulder, CO 80309, USA; 6 Department of Physics, Chemistry and Biology, Linköping University, Linköping 581 83, Sweden; 7 School of Biological Sciences, University of Queensland, Saint Lucia, Queensland 4072, Australia; 8 Centre for Applied Water Science, University of Canberra, Canberra, Australian Capital Territory, Australia; 9 National Centre for Ecological Analysis and Synthesis, University of California, Santa Barbara, 735 State Street, Santa Barbara, CA 93101-5504, USA; 10 Bren School of Environmental Science and Management, University of California, Santa Barbara, CA 93101, USA

**Keywords:** marine biodiversity, ecosystem services, multi-layer networks

## Abstract

Valuing, managing and conserving marine biodiversity and a full range of ecosystem services is at the forefront of research and policy agendas. However, biodiversity is being lost at up to a thousand times the average background rate. Traditional disciplinary and siloed conservation approaches are not able to tackle this massive loss of biodiversity because they generally ignore or overlook the interactive and dynamic nature of ecosystems processes, limiting their predictability. To conserve marine biodiversity, we must assess the interactions and impacts among biodiversity and ecosystem services (BD-ES). The scaling up in complexity from single species to entire communities is necessary, albeit challenging, for a deeper understanding of how ecosystem services relate to biodiversity and the roles species have in ecosystem service provision. These interactions are challenging to map, let alone fully assess, but network and system-based approaches provide a powerful way to progress beyond those limitations. Here, we introduce a conceptual multi-layered network approach to understanding how ecosystem services supported by biodiversity drive the total service provision, how different stressors impact BD-ES and where conservation efforts should be placed to optimize the delivery of ecosystem services and protection of biodiversity.

This article is part of the theme issue ‘Integrative research perspectives on marine conservation’.

## 1. Background

Human wellbeing depends on marine biodiversity. Diverse and healthy marine ecosystems play a fundamental role in the global climate system and in supporting communities, jobs and livelihoods, food security, human health, economic prosperity and a good quality of life [1]. However, many stressors threaten marine life and the services that species support [2,3]. Illegal, unreported and unregulated fishing and overexploitation of fish stocks threaten entire species and food security [4]. Ocean warming, acidification, rising sea levels, pollution and development are expected to accelerate with severe consequences for marine biodiversity [5].

A fundamental question facing society is: how do we manage marine ecosystems to protect both biodiversity and the ecosystem services on which society relies? A major societal challenge of the current century is to ensure a sustainable provision of essential ecosystem services. This includes provisioning (e.g. food security), regulating (e.g. flood and climate regulation) and cultural (e.g. recreational and spiritual wellbeing) services, all of which are confronted by a growing human population and unprecedented rates of biodiversity loss [6].

Since the publication of the Millennium Ecosystem Assessment [7], the importance of valuing the full range of ecosystem services in the context of biodiversity (all plant and animal life) has been among the top priorities in research and policy [1,8,9]. However, there is a gap of sufficient, mechanistic and reliable knowledge about the direct link between biodiversity and ecosystem services (BD-ES). Single species can provide multiple services arising from their different functional traits. For example, mangrove trees sequester carbon, provide shoreline protection, serve as a nursery for key commercial fish stocks and provide fuel for people. Each of these services arises from different functional mangrove traits. But scaling up from single species to entire communities is necessary and challenging: we must understand how ecosystem services emerging from the diversity of traits embedded in biodiversity drive the total service provision. Unfortunately, for too many services in most ecosystems, details of the roles played by single species are poorly understood, and, more critically the effects of species' interdependencies on ecosystem structure, function and service provision are often ignored [10]. Theory predicts that direct and indirect interactions between species can constrain or enhance ecosystem structure, function and service provision in natural ecosystems [11]. Ignoring such interdependencies is risky because they define our ability to forecast how species/biodiversity loss will impact current and future ecosystem service provisioning, how these interdependencies impact human wellbeing, how these interdependencies are constrained by threats and how threats can be mitigated by conservation actions and strategies [12–14].

Here, we propose a multi-layered network approach that will advance the identification of the mechanisms that link interactions between biodiversity, ecosystem services, threats, conservation actions and ultimately human wellbeing. Together, this proposed approach can advance the state-of-the-art understanding of how ecosystem services emerge from, depend on, and are sustained by biodiversity and, once put into practice, help threat mitigation and conservation planning.

## 2. The multi-layered network approach

Ecological networks are traditionally represented as single layered networks illustrating ecological interactions, for example, trophic interactions between species [15]. These networks of trophic relationships in ecosystems provide complex yet tractable depictions of biodiversity, species interactions, and ecosystem structure and function, where species traits explain patterns in the architecture of natural food webs that underpin the structure, functioning and stability of ecosystems. This framework and understanding then paves the way for community-level management of the most complex natural ecosystems [11,16].

Expanding beyond consideration of only ecological interactions, growing awareness of the increasingly connected world and its complexity has catalysed the fusion of networks from different domains, leading to multi-layered network approaches. Multi-layered networks (also referred as network of networks) have attracted a lot of attention recently [17–19]. Kivelä *et al*. [20] provide a comprehensive summary about different types of multi-layered networks, including multi-modal networks [21], multi-dimensional networks [22], multiplex networks [23] and inter-dependent networks [24,25].

**Figure 1.**
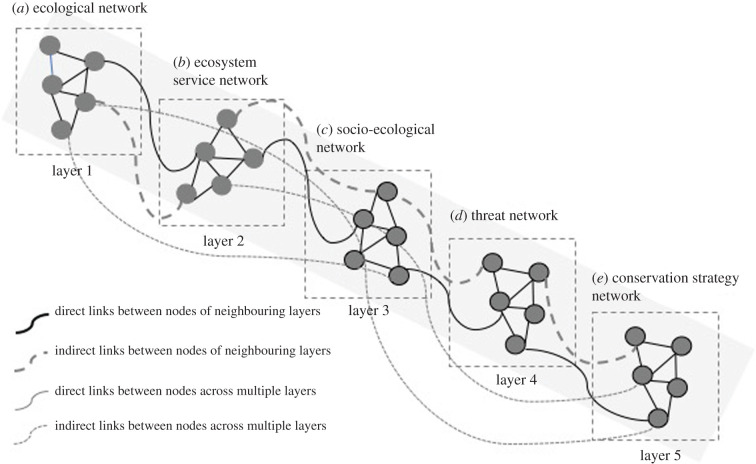
The workflow of the five layers of multi-layered network approach: (*a*) the ecological network, (*b*) the ecosystem service network, (*c*) the socio-ecological network, (*d*) the threat network and (*e*) the conservation strategy network, illustrating the connections within the network, the direct and indirect links between neighbouring and across multiple networks.

One crucial aspect that differentiates multi-layered networks from other network models is their cross-layer dependency, which describes the associations between the nodes from different layers. Where nodes appear in at least one of these layers, these networks are both connected by intra-layer links (links in one layer/network) as well as inter-layer links (links between the nodes of the different layers). For social–ecological systems, multi-layer network approaches are increasing in application to study complex resource management and governance challenges, including in marine systems), by incorporating interactions between and across both social and ecological systems (figure 1) [26].

Where processes endanger individual species directly, it is comparatively simple to understand how best to intervene in a management sense. However, when species are vulnerable owing to both direct threats and indirectly through interactions [27], then a deeper understanding of the ecological network is required. A key point to emerge from thinking about ecosystem service conservation in this way is the need for a holistic systems approach [28,29]. Including network perspectives will allow a genuine understanding of the relationships that may contribute to the vulnerability of the species that underpin the provision of ecosystem services [12]. Analysing the possible consequences of species loss or gain for the provision of ecosystem services at the species level will allow for reducing the risks of service losses and help design conservation plans that using knowledge of which species directly or indirectly affect service delivery and the likelihood that species losses could trigger cascading extinctions by destabilizing networks and entire ecosystems [30,31].

Here, we introduce a multi-layered network approach which builds on ecological network theory to predict consequences of biodiversity loss on ecosystem services and to identify key generalities that simplify the complexity of socio-ecological networks [27,32] and also embrace unique features of these networks allowing derivation of relevant, system-specific and reliable data useful to decision making and policy tools. The multi-layered network approach is set up as a transdisciplinary framework that merges ecosystem-based approaches, ecological network perspectives and dynamic system modelling, which is critical to understand the vulnerability of species and of the ecosystem services they provide.

Adopting a network perspective, relationships are conceptualized as links connecting different nodes which will allow us to construct a multi-layer network to analyse structural patterns of relationships between nodes. We conceptualized this framework as a five-layer network with five types of nodes; species, ecosystem services, social actors, threats and conservation actions, each occupying their own layer (figure 1). This multi-level network thus captures relationships among these five factors, while it also captures synergies and trade-offs within those layers (i.e. which threats interact and may result in cumulative impacts). The inter-layer edges can then represent how biodiversity is tied to ecosystem services [13,14], which services face what kind of threats and what conservation actions can be targeted to either species, services or threat mitigation.

The first layer of the multi-level network approach (figure 1) resembles the ecological network (*a*) of trophic interactions of a given ecosystem (who eats whom). The second layer is the ecosystem service network (*b*), which represents how ecosystem services depend on or impact each other, and this layer links to species (nodes) from the ecological network that contribute to the respective ecosystem services [12]. The third layer represents the social ecological network (*c*), a set of societal actors interact with each other, and also interact/depend on ecosystem services and species in the ecological network [17]. The fourth layer illustrates the threat network (*d*), different stressors interact, i.e. climate change and ocean acidification, and finally the conservation action network (*e*), different conservation strategies have either positive or negative interactions and directly target a service, a species or a threat.

## 3. Discussion

More than three billion people's livelihoods depend on marine and coastal biodiversity, they are important to mitigate climate change, enable cleaner energy, facilitate trade and create jobs. They will be crucial to achieve transformative change [1] as scientists, policy makers and other end-users attempt to address and mitigate anthropogenic pressures, global change and biodiversity loss. Turning the tide of biodiversity loss will require bold and innovative action, as all species are connected to others through ecological interactions, interactions between ecosystem services and synergies among threatening processes often amplify their effects, producing large and accelerating combined impacts. Policy responses and actions tend to tackle threatening processes separately and are, therefore, often not appropriately scaled to interacting threats [33,34].

**Figure 2.**
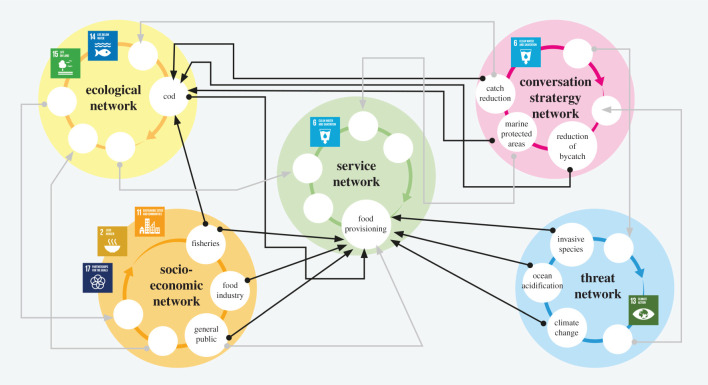
Illustration of the conceptual workflow of the multi-layered network approach using cod and its role in multiple networks as an example, including the identification of the networks as well as the interactions between them. Cod play an essential role in marine ecosystems. As the top predator, cod is of major importance to marine plant and animal life. This means that threatening impacts on the cod stock have consequences throughout the entire ecological network layer, impacting the services cod provides and thereby influencing the socio-ecological network. The cod's crucial role illuminates the importance of fisheries management and conservation strategies that views the multi-layered network as a whole.

The multi-layer network approach (figure 2) can be a transdisciplinary and cross-ecosystem approach to develop the mechanistic knowledge of how economic, organizational and political structures impact on the success and failure of efforts to conserve the relationships between BD-ES. Achieving this is challenging, in part owing to a lack of a comprehensive evidence base that covers all aspects of the question. There is currently insufficient synthesis of how individual species impact ecosystem functions and services. Similarly, the impacts of societal actors on the success or failure of conservation measures are infrequently measured, and certainly not alongside biological information [27,35]. However, there is robust theory about the distribution and magnitude of direct and indirect effects in all kinds of networks, which provides the template for understanding when and how constraints or synergies for ecosystem services arise [13,14].

The multi-layered network approach can provide robust information on how threat-induced feedbacks propagate through ecological and socio-economic networks, and how they vary across spatial scales and through time. Synthesizing biological and socio-economic approaches, creates a critical evidence base of where conservation actions should be targeted and succeed or fail in different contexts [34].

The major ambition of the multi-layered network approach is to advance biodiversity assessment and resource management beyond the traditional single-species abundance approaches on which they remain based, and to provide a framework that instead acknowledges the clear importance of interactions, interdependencies and feedbacks within ecosystems undergoing environmental and human-induced change. However, to achieve this vision, more connection between theoretical approaches and empirical validation of their predictions and recommendations are needed. Existing network models used to support management (particularly in fisheries) contain many untested assumptions, and theoretical approaches typically remain disconnected from the dynamics of real ecosystems and linkages with ecosystems services and socio-economic dynamics [36–38].

The multi-layered network approach will provide a unique, truly integrated ecological approach. While much of current research on the functional role of biodiversity is system-specific, understanding the unifying principles linking diversity to ecosystem services and their stability in natural ecosystems requires a cross-system approach. The extent to which ecosystem properties and dynamics generalize across marine systems is a fundamental question in marine conservation [39,40]. A key ambition of the multi-layered network approach is to allow the identification of ecosystem-specific signatures in the functioning of biodiversity, and cross-ecosystem general patterns.

### (a) Inclusive networks of interactions—from individual interactions to people

Tackling multiple networks of interactions is crucial for understanding and predicting the response of ecological communities to perturbations and their consequences on multiple ecosystem services. It is important not only because we need to consider the whole complexity of ecological networks but also because different services can rely on different interactions (e.g. trophic interactions, pollination, parasitism). Yet ecologists have traditionally studied networks of different interaction types in isolation, and have focused primarily on food webs. Using the multi-layered network approach allows the integration of non-trophic interactions with traditional food web studies and of mutualistic and antagonistic interactions in socio-ecological networks [27].

### (b) Holistic understanding—consequences and causes of multiple drivers of change

The pursuit of society's needs and demands is placing unprecedented pressure on natural resources. The major societal challenge of understanding the interactions between drivers of environmental change, including population growth, economic activities, consumption, urbanization, trade, conflict and governance, is all under the influence of climate change. With the multi-layered network approach, we provide an integrated assessment of impacts on BD-ES provisioning to the multiple stressors and their causes.

The contribution of specific species to ecosystem functions and services is critically dependent on how they are embedded in the community by their interactions. For instance, functions such as primary and secondary production depend not only on the distribution of individual body sizes across species, but also on the trophic interactions that shape top-down control and bottom-up energy fluxes. The innovative and ambitious ecological network-based approach can be used to quantify how species interactions drive community and ecosystem-level response variables such as primary production and other services.

### (c) Connection of processes across multiple scales—advancing predictive complex socio-ecological models

The multi-layered network approach will advance the current understanding of biodiversity and ecosystem functioning, which derives from controlled local settings, to larger spatial and temporal scales by explicitly including dimensions at all relevant scales. This innovative multiplex-network (or meta-network) approach recognizes that multiple networks of interactions (species-networks, trait-networks, spatial-networks) act together in real systems, so understanding how processes scale across spatial, temporal and functional networks is crucial. This constitutes a major advance on much past ecosystem ecology research which has been conducted on a single (increasing) axis of complexity, e.g. scaling from population to community and food-web dynamics, or scaling from local to regional dynamics. The multi-layered network approach will thus uniquely enable us to understand the cascading and cross-scale dynamics that may be crucial to understand the long-term persistence and functionality of natural ecosystems and achieve an integration by a common description and recoding of variables to be linked and scaled in an ecological multiplex-network. This approach will not only benefit ecological research, but can be directly used to understand and analyse socio-economic networks of various kinds, such as combining transportation, disease and communication networks. The multi-layered network approach will also allow for the appropriate integration of cultural services with diverse kinds of values, often neglected in economic valuations [41,42]. We recommend that future empirical work tests the multi-layer network approach we propose here and extends this approach to analyse how interactions of societal actors influence contributions to sustainability.

## 4. Conclusion

As species decline, the resilience of marine ecosystems is reduced, which can in turn lead to an accelerating reduction in biodiversity, ecosystem function and service provision. Effective BD-ES conservation is critical to achieving sustainable development in the face of global change. As such, it needs to be integrated into all sectors and across sectors, resulting in an entangled web of interactions and feedbacks, which complicate management decisions and conservation strategies [29,43,44]. The multi-layered network approach we propose here uses network theory to assess the importance of interactions between biodiversity, people and threats, disentangling the synergies and trade-offs enabling better-informed conservation actions and decisions that can protect species and the services on which society relies.

## Data accessibility

Our paper has no data.
